# ﻿Two new species of the genus *Terrobittacus* Tan & Hua, 2009 (Mecoptera, Bittacidae)

**DOI:** 10.3897/zookeys.1148.97997

**Published:** 2023-02-14

**Authors:** Le-Le He, Bao-Zhen Hua

**Affiliations:** 1 Entomological Museum, College of Plant Protection, Northwest A&F University, Yangling, Shaanxi 712100, China Northwest A&F University Yangling China

**Keywords:** Biodiversity, China, hangingflies, Oriental region, *Terrobittacus*

## Abstract

Two new species of *Terrobittacus* Tan & Hua, 2009 are described and illustrated from Sichuan and Guangxi provinces of China, increasing the species number of *Terrobittacus* to eight. *Terrobittacusemeishanicus***sp. nov.** is differentiated from its congeners by wings with distinct markings and a female subgenital plate with a V-shaped carina. *Terrobittacuslaoshanicus***sp. nov.** can be recognized by the black terga VI–IX. A key to species of *Terrobittacus* is updated. The species distribution and the relationship between adult morphology and mating behavior were briefly discussed.

## ﻿Introduction

The hangingfly genus *Terrobittacus* Tan & Hua, 2009, a small taxon of Bittacidae Handlirsch, 1906 from the Oriental China, was established with *Bittacusimplicatus* Huang & Hua, 2006 as its type species, with six known species to date (Du and Hua 2017). The genus can be recognized by the male epandrial appendage shorter than half the length of the gonocoxites; aedeagal lobe small and acute; dorsal portion of tergum X absent or strongly vestigial into a narrow transverse plate; and the two halves of the subgenital plate almost fused in the female (Tan and Hua 2009).

The ovary of *Terrobittacus* consists of seven polytrophic ovarioles, and each vitellarium consists of five or six egg chambers, which comprises three nurse cells and one oocyte (Yan 2018). Spermiogenesis and sperm ultrastructure support a reversal origin of the 9 + 2 flagellar axoneme in Mecoptera (Miao et al. 2019). The vasa deferentia differ considerably from those of Panorpidae (Lyu and Hua 2019). *Terrobittacus* possesses the highest chromosome number ever observed in Bittacidae and an asymmetric karyotype (Miao and Hua 2017).

The eggs are characterized by the globe shape with a grid of latitudes on the chorion (Tan and Hua 2009). The larvae are eruciform, bearing a pair of prominent lateral compound eyes of seven ommatidia on the head (Zheng et al. 2022). The six elongate Malpighian tubules of larvae contain abundant spherites, which are likely associated with the interesting habit of soil-spraying (Liu and Hua 2018). The fine structure and functional morphology of adult mouthparts are well documented (Ma et al. 2014).

In this paper, two new species of *Terrobittacus* are described and documented, increasing the number of *Terrobittacus* species to eight. The key to species of *Terrobittacus* is also updated.

## ﻿Materials and methods

### ﻿Sampling

Adult specimens were collected from the mountain regions in Guangxi and Sichuan provinces of China. The specimens used in this study are deposited in 75% ethanol at the Entomological Museum, Northwest A&F University, China (**NWAU**).

### ﻿Morphological observations

Specimens were observed under a Nikon SMZ1500 microscope. Habitus photographs were taken with a Nikon D7100 digital camera and character pictures were taken using a scientific digital micrography system ZEISS SteREO Discovery V20, equipped with an automontage imaging system (AxioCam IC). All photographs were assembled with Adobe Photoshop 2022. The measurements were obtained with a vernier caliper and are presented as mean ± SD (standard deviation).

### ﻿Acronym definitions

Terminology follows Zhang et al. (2020). The following abbreviations are applied in the measurements: **AL** antennal length; **BL** body length; **FL** forewing length; **FW** forewing width; **HL** hindwing length; **HW** hindwing width.

The following abbreviations are used in the figures: **A** anal vein; **AL** aedeagal lobe; **Av** apical cross-vein between CuP and 1A; **Ce** cercus; **Cly** clypeus; **CuA** anterior cubitus; **CuP** posterior cubitus; **Cuv** apical cross-vein between CuA and CuP; **EA** epandrial appendage; **FM** fork of media; **Fr** frons; **FRs** first fork of radial sector; **Gcx** gonocoxite; **Gs** gonostylus; **L** labrum; **LBP** lower branch of proctiger; **LP** labial palp; **M** media; **MP** maxillary palp; **OM** origin of media; **ORs** origin of radial sector; **Pcv** cross-veins between R_1_ and R_2_ behind the pterostigma; **Pf** penisfilum; **Ps** pterostigma; **R_1_** first radius; **S** sternum; **SaP** subanal plate; **Sc** subcostal vein; **Scv** cross-vein between distal half of Sc and R_1_; **SgP** subgenital plate; **Sp** spiracle; **T** tergum; **UBP** upper branch of proctiger.

## ﻿Taxonomy

### ﻿ Terrobittacus emeishanicus sp. nov.

Taxon classificationAnimaliaMecopteraBittacidae

6EBE1E6C-4353-59FC-BC9E-56BFAB5D0C2B

https://zoobank.org/DFFEBE84-C3F2-4AB0-A103-0AD12FE9B8D3

Figs 1 , 2

#### Type material.

***Holotype***: ♂; China, Sichuan Province, Emeishan; 29°35'10"N, 103°11'19"E; alt. 1320 m; 28 July 2021; leg. Lu Liu, Jia-Yi Ren and Jie Zhang. ***Paratypes***: 3♂11♀, same data as for the holotype.

#### Diagnosis.

The new species can be readily recognized from its congeners by the following characters: 1) wing with numerous markings and a reddish brown pterostigma; 2) femora and tibiae apices, and hind legs tarsi reddish brown; 3) male epandrial appendages tapering toward apex; 4) male cerci clavate, slightly expanded distally; 5) gonocoxites with one to three pairs of long brown distal bristles; and 6) basal half of female subgenital plate fused, with a black, strongly sclerotized V-shaped carina along midventral line, distal half cleft.

#### Description.

***Measurements*** (*N* = 15): AL = 7.62 ± 0.49 mm; BL = 15.66 ± 1.00 mm; FL = 22.26 ± 0.53 mm, FW = 5.08 ± 0.21 mm; HL = 19.55 ± 0.50 mm, HW = 4.69 ± 0.23 mm.

**Figure 1.**
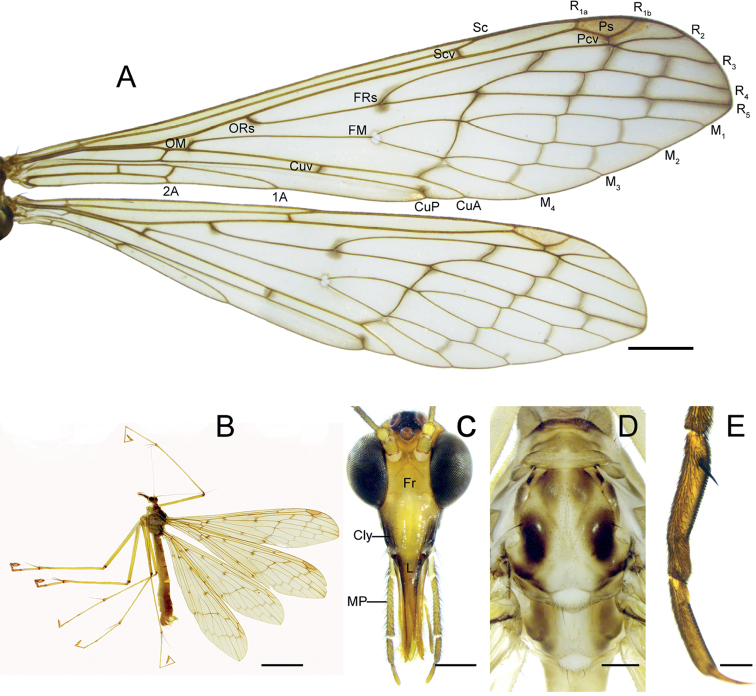
*Terrobittacusemeishanicus* sp. nov. **A** right wings of male **B** male habitus in lateral view **C** head in frontal view **D** thorax in dorsal view **E** tarsomeres IV, V and claw of left foreleg. Abbreviations: see Materials and methods. Scale bars: 2.0 mm (**A**); 5.0 mm (**B**); 0.5 mm (**C, D**); 0.2 mm (**E**).

***Head*** (Fig. 1C) Vertex and frons yellowish brown; ocellar triangle black, lateral ocelli twice as large as median ocellus; clypeus and labrum yellowish to blackish brown, lateral sides darker; maxillary palp yellowish brown, 3^rd^ segment longer than 4^th^ and 5^th^ segments combined. Antennae yellowish brown; scape cylindrical; pedicel spherical; flagellum filiform and ciliated, with distinct segments basally and obscure beyond 13^th^ segment.

***Thorax*** (Fig. 1D) Nota unevenly blackish brown; mesonotum prominence darker, each with one black seta; meso- and metanotum with light median streak, bearing one or two setae on posterior margins. Pleura, coxae and mera light brown. Legs yellowish brown; femora and tibiae apices, and hind leg tarsi reddish brown (Fig. 1B); hind basitarsus as long as tarsomeres II–IV together; one or two black spines along each side of proximal tarsomere IV (Fig. 1E).

***Wings*** (Fig. 1A) Forewing membrane hyaline with yellowish tinge; pterostigma reddish brown; grayish brown clouding flecks at OM, ORs, FRs, FR_4+5_, CuP ending, and most cross-veins; Pcv one. FRs near FM; Sc ending slightly beyond level of FR_4+5_; Scv beyond FRs; CuP ending posteriorly curved, and near FM_3+4_; Cuv before level of FM; 1A ending before FM; 2A ending near OM; Av absent. Hindwing similar to forewing in general pattern and coloration, but Sc ending and Scv before level of FRs.

**Figure 2.**
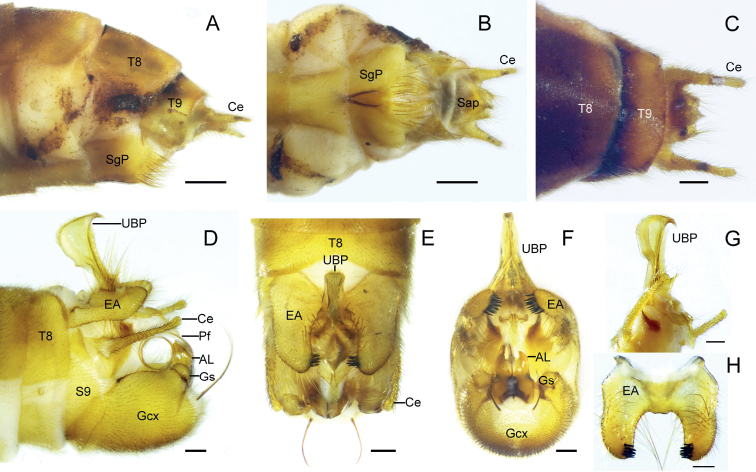
*Terrobittacusemeishanicus* sp. nov. **A** female terminalia in lateral view **B** female terminalia in ventral view **C** female terminalia in dorsal view **D** male terminalia in lateral view **E** male terminalia in dorsal view **F** male terminalia in posterior view **G** abdominal segment X and proctiger in lateral view **H** epandrial appendages in ventral view. Abbreviations: see Materials and methods. Scale bars: 0.5 mm (**A, B**); 0.2 mm (**C–H**).

***Abdomen of male*** (Fig. 2D–H) Terga II–VIII yellowish brown, gradually darker, each with a black antecosta; tergum VIII emarginate in V-shaped on posterior margin. Epandrial appendages yellowish brown, equal or shorter than half length of gonocoxites, quadrangular in lateral aspect, with long yellow hairs along margins; basal portion broad, tapering toward apex (Fig. 2H); bearing sparse long yellow hairs and nine to eleven stout black apical spines on inner surface. Dorsal part of tergum X greatly vestigial, ventral plate yellowish to reddish brown, extending from cercus to base of proctiger. Upper branch of proctiger sickle-like, posteriorly curved; anterior central edge slightly constricted; bearing two small hairy denticulate processes basally; lower branch of proctiger short, tapering toward apex (Fig. 2G). Cerci clavate, slightly expanded at distal, shorter than gonocoxites. Gonocoxites yellowish brown, with one to three pairs of prominently long brown distal bristles. Gonostylus short, irregular-shaped, and bearing numerous brown setae. Basal aedeagal lobes broad, distal portions slender and acute; penisfilum greatly coiled.

***Abdomen of female*** (Fig. 2A–C) Terga II–IX yellowish brown, each with a black antecosta. Subgenital plate sclerotized, yellowish brown; basal half fused, with a black, strongly sclerotized V-shaped carina along midventral line; distal half bearing numerous brown setae and divided mesially by membrane. Tergum IX slightly truncated distally. Tergum X extending ventrad and beyond base of cerci. Supraanal plate tapering toward apex; subanal plate broad, almost truncated apically, shorter than supraanal plate. Cerci slender, longer than anal plates.

#### Etymology.

The specific epithet refers to the type locality, Emeishan.

#### Distribution.

China (Sichuan Province).

#### Remarks.

As far as we know, *Terrobittacusemeishanicus* sp. nov. is probably the largest species of the genus, at least in terms of wingspan. The new species is similar to *T.longisetus* Tan & Hua, 2009 in body color and male genitalia, but can be differentiated from the latter by following features: numerous prominent markings on the wing (cf. no prominent markings); male epandrial appendages tapering toward the apex (cf. male epandrial appendages broad); male cerci slightly expanded distally (cf. thickening toward apex); and a V-shaped carina along the midventral line of the female subgenital plate (cf. X-shaped).

### ﻿ Terrobittacus laoshanicus sp. nov.

Taxon classificationAnimaliaMecopteraBittacidae

299DEC3E-7C68-5B94-8256-21D70656ECA6

https://zoobank.org/63D20DE0-6AA5-473C-8E73-6BEDFB9C6013

Figs 3 , 4 , 5

#### Type material.

***Holotype***: ♂; China, Guangxi Province: Tianlin County, Laoshan Forest Farm; 24°23'51"N, 106°23'9"E; alt. 1270 m; 24–29 June 2022; leg. Le-Le He and Ya-Long Li (NWAU). ***Paratypes***: 3♂7♀, same data as for the holotype.

#### Diagnosis.

This new species is distinguishable from its congeners by the basal half of mesonotum unevenly blackish brown, distal half yellowish brown; terga II–V yellowish brown, terga VI–IX black; the epandrial appendage triangular, with a tooth on basal ventral margin; the gonostylus with a process on inner side; female subgenital plate almost completely fused.

#### Description.

***Measurements*** (*N* = 11): AL = 4.77 ± 0.42 mm; BL = 11.19 ± 1.28 mm; FL = 14.51 ± 0.56 mm, FW = 3.51 ± 0.11 mm; HL = 12.44 ± 0.38 mm, HW = 3.05 ± 0.21 mm.

**Figure 3.**
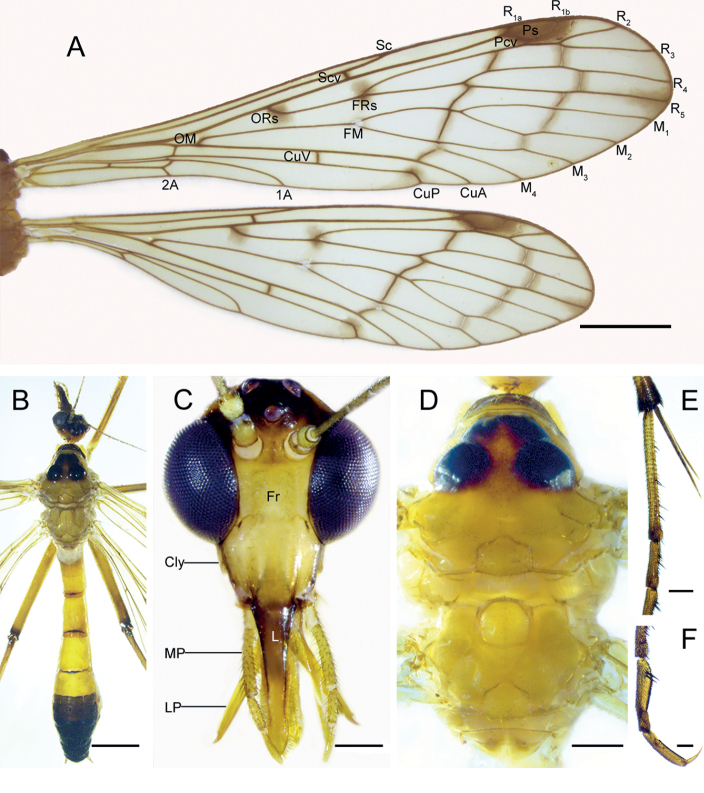
*Terrobittacuslaoshanicus* sp. nov. **A** right wings of male **B** female body in dorsal view **C** head in frontal view **D** thorax in dorsal view **E** tibia with two apical tibial spurs of left foreleg **F** tarsomeres IV, V and claw of left foreleg. Abbreviations: see Materials and methods. Scale bars: 2.0 mm (**A, B**); 0.5 mm (**C, D**); 0.2 mm (**E, F**).

***Head*** (Fig. 3C) Vertex and frons yellowish brown; ocellar triangle black, dark ocellar strip extending to compound eyes; clypeus yellowish brown; labrum unevenly blackish brown; maxillary palp yellowish brown, 3^rd^ segment equal to 4^th^ and 5^th^ segments combined. Antennae yellowish brown; scape and pedicel spherical; flagellum filiform and ciliated, with distinct segments basally and obscure beyond 10^th^ segment.

***Thorax*** (Fig. 3D) Pronotum blackish brown; anterior half of mesonotum unevenly blackish brown, posterior half mesonotum and metanotum yellowish brown. Pleura, coxae and mera light brown (Fig. 5A). Legs yellowish to reddish brown; femora and tibiae with distinct blackish brown apices; length of two apical tibial spurs almost equal (Fig. 3E); hind basitarsus as long as tarsomeres II–IV together; tarsomere IV with two black spines along each side (Fig. 3F).

***Wings*** (Fig. 3A) Forewing membrane hyaline with yellowish brown tinge; pterostigma reddish brown; four conspicuous markings each at ORs, FRs, CuP ending, and R_5_ ending; remaining cross-veins with diffuse clouding flecks; Pcv one. FRs near level of FM; Sc ending distantly before level of FR_4+5_; Scv near FRs; CuP ending curved posteriorly, and near FM_3+4_; Cuv before level of FM; 1A ending before FM; 2A ending before OM; Av absent. Hindwing similar to forewing in general pattern and coloration, but Sc ending before level of FRs.

**Figure 4.**
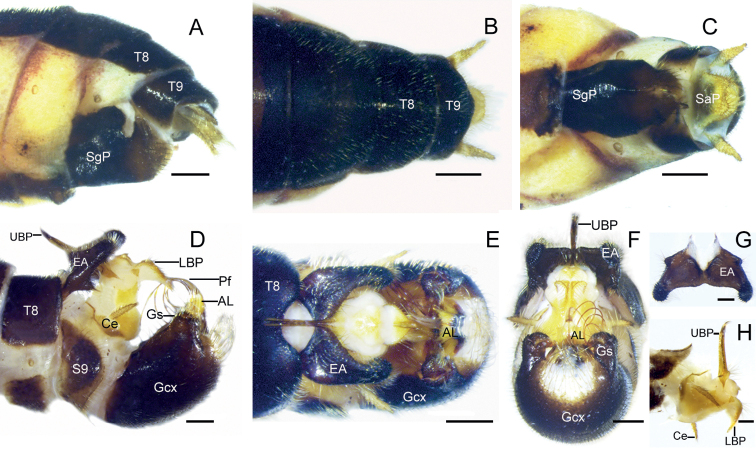
*Terrobittacuslaoshanicus* sp. nov. **A** female terminalia in lateral view **B** female terminalia in dorsal view **C** female terminalia in ventral view **D** male terminalia in lateral view **E** male terminalia in dorsal view **F** male terminalia in posterior view **G** epandrial appendages in ventral view **H** abdominal segment X and proctiger in lateral view. Abbreviations: see Materials and methods. Scale bars: 0.5 mm (**A–C, E**); 0.2 mm (**D, F–H**).

***Abdomen of male*** (Fig. 4D–H) Terga II–V yellowish brown, each with a black antecosta; terga VI–IX black (Fig. 3B). Tergum VIII emarginate on posterior margin. Epandrial appendage triangular, prominently shorter than half length of gonocoxites; distal inner surface bearing a cluster of more than 50 black spines (Fig. 4G), with a tooth on basal ventral margin. Tergum X greatly vestigial, narrow brown lateral plate extending to base of cercus. Upper branch of proctiger yellowish brown, long and straight, with hairy apex; lower branch of proctiger short, tapering toward apex, curved ventrad (Fig. 4H). Cerci yellowish brown, considerably short, about one-quarter length of gonocoxites, acute apically. Gonocoxites blackish brown and rounded, distal membranous area furnished with numerous long yellow hairs. Gonostylus short, with process on inner side, and surrounded by sparse brown setae. Aedeagal lobes broad basally, with two small acute apexes; penisfilum coiled into loops.

***Abdomen of female*** (Fig. 4A–C) Abdomen similar to that of male in coloration. Subgenital plate sclerotized, blackish brown, two halves almost completely fused, several brown setae along distal portion. Tergum X narrow, not extending ventrad. Supra- and subanal plates yellowish brown, broad; almost equal length. Cerci yellowish brown, tapering toward apex, longer than anal plates.

#### Etymology.

The specific epithet refers to the type locality, Laoshan Forest Farm.

#### Distribution.

China (Guangxi Province).

#### Remarks.

The new species resembles *T.echinatus* (Hua & Huang, 2008) in wing coloration and pattern, but can be readily differentiated from the latter by the following traits: terga II–V yellowish brown, terga VI–IX black (cf. terga II–IX yellowish brown); female subgenital plate almost completely fused (cf. cleft by membrane); epandrial appendages triangular (cf. roughly trapezoid).

### ﻿Key to species of *Terrobittacus* (modified from Du and Hua 2017)

**Table:** 

1	Wing with FM_1+2_ slightly before level of FR_4+5_; cerci equal to or longer than epandrial appendages; gonocoxites with one to three pairs of crossed long distal bristles	**2**
–	Wing with FM_1+2_ beyond level of FR_4+5_; cerci shorter than epandrial appendages; gonocoxites without paired crossed long distal bristles	**3**
2	Wing with grayish brown markings at OM, ORs, FRs, FR_4+5_, CuP ending, and most cross-veins; male epandrial appendages tapering toward apex; V-shaped carina along midventral line of female subgenital plate	***T.emeishanicus* sp. nov.**
–	Wing without distinct markings; male epandrial appendages broad; X-shaped carina along midventral line of female subgenital plate	***T.longisetus* Tan & Hua, 2009**
3	Distinct markings diffused along most cross-veins	**4**
–	No distinct markings along cross-veins	**5**
4	Terga II–V yellowish brown, terga VI–VIII black; male epandrial appendages roughly triangular in lateral view; female subgenital plate almost completely fused	***T.laoshanicus* sp. nov.**
–	Terga II–VIII yellowish brown; male epandrial appendages roughly trapezoid in lateral view; female subgenital plate cleft by membrane	***T.echinatus* (Hua & Huang, 2008)**
5	Forewing with two transverse rows of cross-veins in radial and medial sectors; proctiger slender, sabre-shaped, curved caudad, acute apically	***T.xiphicus* Tan & Hua, 2009**
–	Forewing with three transverse rows of cross-veins in radial and medial sectors; proctiger relatively thick; apex curved caudoventrally into a hook, like rostrum of a parrot	**6**
6	Dark ocellar strip extending to compound eyes; male epandrial appendages narrow in lateral view; middle part of female subgenital plate fused	***T.angustus* Du & Hua, 2017**
–	Dark ocellar strip not extending to compound eyes; epandrial appendages broad in lateral view; female subgenital plate cleft by a narrow membranous line mesially	**7**
7	Forewing with Sc ending beyond level of FR_4+5_; epandrial appendages boot-shaped in lateral view	***T.rostratus* Du & Hua, 2017**
–	Forewing with Sc ending before level of FR_4+5_; epandrial appendages triangular in lateral view	***T.implicatus* (Huang & Hua, 2006)**

## ﻿Discussion

**Figure 5.**
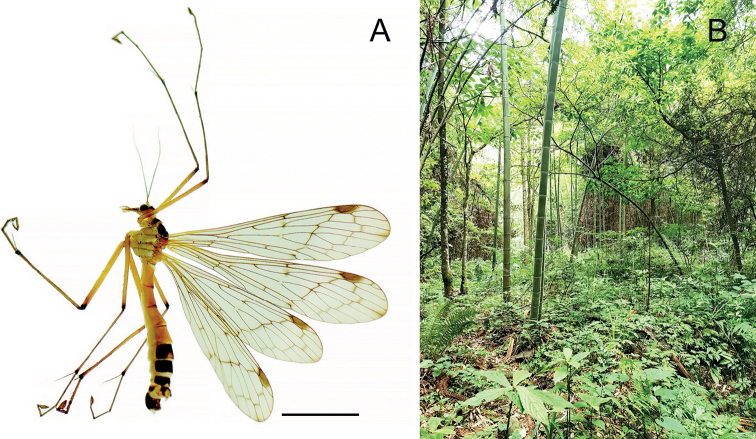
*Terrobittacuslaoshanicus* sp. nov. **A** male habitus in lateral view **B** habitat. Scale bar: 5.0 mm (**A**).

The genus *Terrobittacus* is endemic to China (Tan and Hua 2009). According to the collecting records, the species of *Terrobittacus* are only distributed in the high-altitude microhabitats of cool and humid forests (Fig. 5B), a typical cool-adapted group of insects. The species of *Terrobittacus* have been recorded from Fujian, Guizhou, Henan, Hubei, Hunan, and Shaanxi provinces (Du and Hua 2017). This is the first discovery of *Terrobittacus* in Guangxi and Sichuan provinces.

Male hangingflies use their epandrial appendages to grasp the female abdomen for maintaining a unique belly-to-belly mating position during copulation (Gao and Hua 2013). The male epandrial appendages of *Terrobittacus* are smaller and shorter compared with those of other confamilial genera, resulting in clamping a very small area on the female abdomen. Wei et al. (2020) speculated that smaller epandrial appendages may bear more spines to increase friction. The female abdomen may receive more force on a smaller area so that the degree of sclerotization of the female subgenital plate becomes stronger.

The following phenomena observed in this study may support the hypothesis of Wei et al. (2020). The triangular epandrial appendage of *Terrobittacuslaoshanicus* sp. nov. is narrow and small, bearing more than 50 spines on the inner surface, and the female subgenital plate is almost completely fused and strongly sclerotized. Nevertheless, the quadrangular epandrial appendage of *T.emeishanicus* sp. nov. is broad and longer, bearing only about 10 spines on the inner surface. Correspondingly, the basal half of the female subgenital plate is fused and sclerotized, whereas the distal half is divided mesially by a membrane. In this case, *Terrobittacus* likely provides vital material for studying sexually antagonistic coevolution of insects.

## Supplementary Material

XML Treatment for Terrobittacus emeishanicus

XML Treatment for Terrobittacus laoshanicus
